# Physiological impact of *in vivo* stable isotope tracing on cancer metabolism

**DOI:** 10.1016/j.molmet.2021.101294

**Published:** 2021-07-10

**Authors:** Manuel Grima-Reyes, Adriana Martinez-Turtos, Ifat Abramovich, Eyal Gottlieb, Johanna Chiche, Jean-Ehrland Ricci

**Affiliations:** 1 Université Côte d’Azur, INSERM, C3M, Nice, France; 2 Equipe labellisée LIGUE Contre le Cancer, Nice, France; 3 Ruth and Bruce Rappaport Faculty of Medicine, Technion – Israel Institute of Technology, Haifa, Israel

**Keywords:** Stable isotope tracing, Tracer administration, Interorgan exchange, Fasting, Tumor metabolism

## Abstract

**Background:**

There is growing interest in the analysis of tumor metabolism to identify cancer-specific metabolic vulnerabilities and therapeutic targets. Finding of such candidate metabolic pathways mainly relies on the highly sensitive identification and quantitation of numerous metabolites and metabolic fluxes using metabolomics and isotope tracing analyses. However, nutritional requirements and metabolic routes used by cancer cells cultivated *in vitro* do not always reflect the metabolic demands of malignant cells within the tumor milieu. Therefore, to understand how the metabolism of tumor cells in its physiological environment differs from that of normal cells, these analyses must be performed *in vivo*.

**Scope of Review:**

This review covers the physiological impact of the exogenous administration of a stable isotope tracer into cancer animal models. We discuss specific aspects of *in vivo* isotope tracing protocols based on discrete bolus injections of a labeled metabolite: the tracer administration *per se* and the fasting period prior to it. In addition, we illustrate the complex physiological scenarios that arise when studying tumor metabolism – by isotopic labeling in animal models fed with a specific amino acid restricted diet. Finally, we provide strategies to minimize these limitations.

**Major Conclusions:**

There is growing evidence that metabolic dependencies in cancers are influenced by tissue environment, cancer lineage, and genetic events. An increasing number of studies describe discrepancies in tumor metabolic dependencies when studied in *in vitro* settings or *in vivo* models, including cancer patients. Therefore, in-depth *in vivo* profiling of tumor metabolic routes within the appropriate pathophysiological environment will be key to identify relevant alterations that contribute to cancer onset and progression.

## Highlights

• *In vivo* isotope tracing is the state-of-the-art approach to study tumor metabolism.
• *In vivo* tracer administration challenges the physiological metabolism of mice.
• Interorgan conversion of the tracer might confound tumor labeling patterns.
• Mouse fasting before *in vivo* tracing impacts on systemic and tumor metabolism.
• Optimization is key to minimize physiological alterations linked to *in vivo* tracing.

## 1 Introduction

Metabolic reprogramming has been recognized as a hallmark of cancer [1,2]. The challenge of expanding our understanding of major cancer metabolic features and specific metabolic dependencies requires sophisticated approaches, such as metabolomics. Metabolomics allows the identification and relative quantitation of numerous metabolites by mass spectrometry coupled to gas or liquid chromatography (GC/LC-MS). Other techniques such as magnetic resonance spectroscopy enable metabolite identification and quantification, but at a lower scale [3]. Metabolomics endows cancer researchers with a high-resolution tool for the quantification of absolute and relative abundances of metabolite pools in malignant tissues and biofluids surrounding the tumoral mass [4]. Levels of these small molecules provide hints of which metabolic pathways have been aberrantly altered during oncogenic transformation [5].

**Figure 1:**
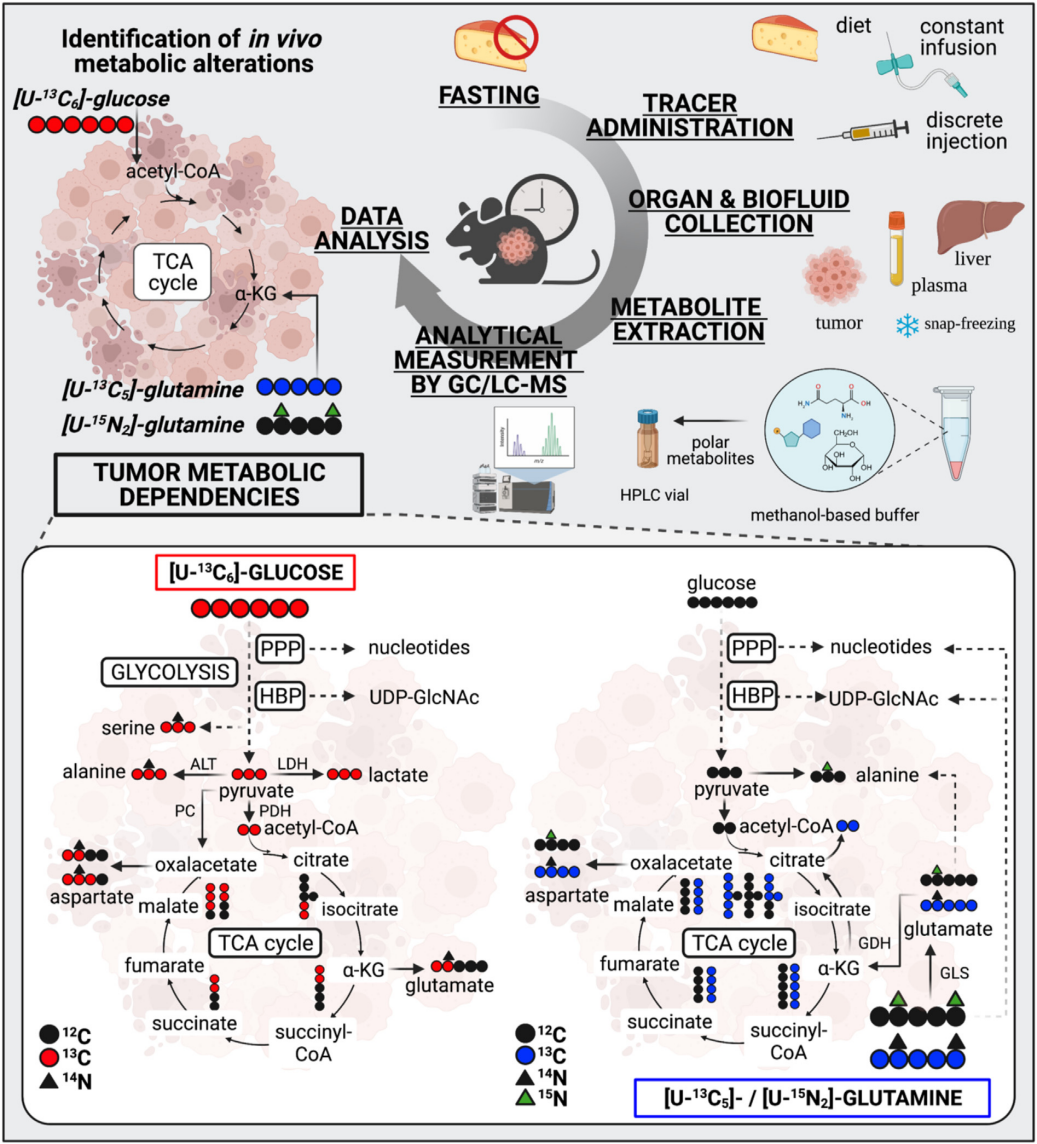
**Time course steps of *in vivo* stable isotope tracing approaches**. Lower panel, carbon, and nitrogen fate of labeled glucose and glutamine in main metabolic pathways. PPP, pentose phosphate pathway; HBP, hexosamine biosynthetic pathway; TCA, tricarboxylic acid cycle; ALT, alanine aminotransferase; LDH, lactate dehydrogenase; PDH, pyruvate dehydrogenase; PC, pyruvate carboxylase; GLS, glutaminase; and GDH, glutamate dehydrogenase. Created with BioRender.com.

Stable isotope resolved metabolomics allows to monitor how labeled metabolic sources contribute to bioenergetic, biosynthetic, and/or redox pathways that sustain tumoral tissues in their transformed state (Figure 1). Depending on the intracellular metabolic labeling, isotope tracing helps to infer metabolite interconversion, a feature that cannot be perceived by steady state metabolomics [6]. Tracing the incorporation of stable isotopes of carbon (^13^C), nitrogen (^15^N), or hydrogen (^2^H) from isotopically labeled nutrients (tracers) into downstream tissue metabolites is considered the state-of-the-art approach to study cancer metabolism [7]. This methodology determines the isotopic composition of metabolites based on the differences in atomic masses. For instance, the heavy stable isotope of carbon (^13^C) has a molecular mass increased by a unit (M+1) as compared to the most naturally abundant carbon isotope (^12^C). These differences in nominal masses allow to distinguish the fully labeled glucose with six heavier carbons (M+6) from unlabeled glucose (M+0). Therefore, tracing a stable labeled nutrient into downstream metabolites allows to follow the cascade of chemical reactions by which nutrient catabolism or anabolism is increased or decreased. Aberrant metabolite uptake or secretion from tissues can also be estimated by isotopic labeling.

Tumors usually reside in a poorly vascularized microenvironment under nutritional conditions that significantly differ from those found in cell culture media [8,9]. Therefore, cancer cells growing *in vitro* do not necessarily display the same metabolic phenotype as the intact tumor. For instance, Ras-driven lung tumors in mice are more dependent on glucose to fuel the tricarboxylic acid (TCA) cycle and less dependent on glutamine compared to their *in vitro* counterpart cell lines [10]. Nutritional availabilities and metabolite exchange between stromal and cancer cells also determine how tumor cells will metabolically adapt to competitively growth and survive [3]. Therefore, *in vivo* tracer-based metabolomics is the most authentic approach for studying the nutritional requirements and metabolic reprograming of intact malignant tissues. For instance, tracing the incorporation of uniformly carbon labeled glucose, ([U–^13^C_6_]-glucose), into downstream metabolites has expanded our notions of how glucose is metabolized in tumoral tissues (Figure 1). However, preferential glucose contribution to the TCA cycle through the activity of pyruvate carboxylase (PC) – as compared to the activity of pyruvate dehydrogenase (PDH) – has been reported in mouse models of lung cancer and breast cancer-derived lung metastasis. The differential labeling patterns of TCA cycle intermediates arising from reactions catalyzed by PC and PDH in the presence of [U–^13^C_6_]-glucose have allowed to estimate the relative activity of the former enzymes for anaplerotic replenishment of the TCA cycle in these tumors [11,12]. *De novo* serine biosynthesis from fully carbon-labeled glucose has been demonstrated to preferentially occur in lung metastases of breast cancer as compared to primary tumors by quantitation of serine M+3 [13]. [U–^13^C_5_]-glutamine fueling the TCA cycle through glutaminase activity has been demonstrated by the labeling pattern of glutamate M+5 and TCA cycle intermediates. The latest metabolic pathway has been shown to preferentially occur in *in vitro* culture of lung tumor cells as compared to lung tumors growing in mice [10]. In addition, KRAS mutant/LKB1 deleted-driven lung cancer cells labeled with ^15^N_1_-amide-glutamine display a high flux through the hexosamine biosynthetic pathway reflected by an increase in the detection of ^15^N-UDP-GlcNAC [14] (also refer to Table 1 for more examples and details). Beyond preclinical research, stable isotope labeling to study cancer metabolism in patients highlighted that glucose does not only yield energy by aerobic glycolysis, but is also terminally oxidized in several types of tumors. Moreover, radioisotope labeled nutrients, such as the glucose analogue ^18^FDG (Fluorodeoxyglucose), are commonly used for cancer diagnosis and follow-up treatment by positron emission tomography [3].

**Table 1:** Compilation of *in vivo* GC/LC-MS-based stable isotope tracing studies using labeled glucose, glutamine, and lactate in cancer mouse models.

Tracer administration	Fasting	Experimental details	Metabolic analysis	Ref
[^13^C]-glucose				
*Ad libitum* liquid diet	None	[U–^13^C_6_]-glucose in liquid diet for 18 h	Fractional enrichment of intra-tumoral metabolites in NSCLC PDX models versus *ex vivo* tissue cultures	[20]
Infusion	Diurnal fasting of 6 h	[U–^13^C_6_]-glucose at 30 and 20 mg/kg/min for 6 h through the jugular vein and carotid artery in free-moving mice, anesthesia for mouse sacrifice	Glucose contribution to the TCA cycle in Ras-driven NSCLC tumors and tumor-derived cell lines	[10]
Infusion	Unspecified	[U–^13^C_6_]-glucose at 30 mg/kg/min for 6 h through the jugular vein	Pyruvate carboxylase activity estimation by differences between malate M+3 and succinate M+3 in breast cancer tumors and derived lung metastases	[12]
Infusion	Unspecified	[U–^13^C_6_]-glucose at 30 mg/kg/min for 6 h through the jugular vein	Proline catabolism by the activity of proline dehydrogenase in breast primary tumors and derived lung metastasis	[49]
Infusion	Nocturnal fasting of 16 h	[U–^13^C_6_]-glucose at approximately 540 mg/kg/min for 1 min by initial bolus followed by 11 mg/kg/min for 3 h through the tail vein under anesthesia [^13^C_3_]-lactate at 36 mg/kg/min for 10 min by initial bolus followed by 6 mg/kg/min for 3 h	Contribution of circulating lactate to glycolytic intermediates and the TCA cycle by co-infusion of fully labeled glucose and positional labeled lactate in tumors of human NSCLC xenografts in mice	[28]
Infusion	Nocturnal fasting	[U–^13^C_6_]-glucose at 1 mg/kg/min (after a 5 min priming) for 2 h through the jugular vein	Contribution of circulating glucose to the TCA cycle in colorectal tumors under anti-metabolic treatments	[50]
Infusion	Fasting of 6 h	[U–^13^C_6_]-glucose at 20 mg/kg/min for 3 h through the jugular vein in free-moving mice	Glucose oxidation and acetate production in primary soft tissue sarcoma mouse models	[51]
Infusion	Fasting of 16 h	[U–^13^C_6_]-glucose and [^13^C_1,2_]-glucose at 412.5 mg/kg for 1 min by initial bolus followed by 8 mg/kg/min for 3 h through the tail vein under anesthesia	Contribution of glucose to pyruvate and lactate Relative glucose flux through glycolysis over the PPP (ratio lactate M+2/M+1)	[52]
Infusion	Unspecified	[U–^13^C_6_]-glucose at 30 mg/kg/min for 6 h through the jugular vein	Glucose contribution to *de novo* biosynthesis of serine in primary breast cancer tumors and derived lung metastases	[13]
Discrete bolus	Unspecified	[U–^13^C_6_]-glucose at approximately 1 g/kg every 15 min for 1 h through the tail vein	Glucose entry to the TCA cycle by pyruvate carboxylase in a NSCLC mouse xenograft	[11]
Discrete bolus	Unspecified	[U–^13^C_6_]-glucose at 1 g/kg for 22 min through the tail vein [U–^13^C_5_]-glutamine at 0.15 g/kg	Glucose contribution to *de novo* synthesis of glutamine by glutamine synthetase in glioblastoma PDX mouse models	[23]
[^13^C]-glutamine and [^15^N]-glutamine				
Infusion	Unspecified	[U–^13^C_5_]-glutamine at 1.9 mg/kg/min for 4 h through the intra-carotid artery under anesthesia	Uptake of circulating glutamine by tumor cells in glioblastoma PDX mouse models	[23]
Infusion	Diurnal fasting of 6 h	[U–^13^C_5_]-glutamine at 2.0 mg/kg/min and 3.7 mg/kg/min for 6 h through the jugular vein and the carotid artery in free-moving mice, anesthesia for mouse sacrifice	Glutamine contribution to the TCA cycle in Ras-driven NSCLC tumors and tumor-derived cell lines	[10]
Infusion	Fasting of 16 h	[U–^13^C_5_]-glutamine at 172.5 mg/kg for 1 min by initial bolus followed by 2.88 mg/min/kg for 5 h through the tail vein under anesthesia	Contribution of glutamine to the TCA cycle intermediates in melanoma PDX mouse models	[52]
Infusion	Nocturnal fasting of 16 h	[γ-^15^N]-glutamine at 300 mg/kg for 1 min by initial bolus followed by 5 mg/kg/min for 5 h through the tail vein under anesthesia	Glutamine γ-nitrogen contribution to the HBP by detection of UDP-HexNAc M+1 in tumors of subcutaneous lung xenograft mouse models	[14]
Discrete bolus	Unspecified	[γ-^15^N]-glutamine at 700 mg/kg for 2 h and 4 h by intra-peritoneal injection	Contribution of glutamine-derived γ-nitrogen to orotate and dihydroorotate synthesis in subcutaneous Hela xenograft and breast tumors	[53]
Discrete bolus	Unspecified	[U–^13^C_5_]-glutamine and [U–^15^N_2_]-glutamine at 100 mg/kg for 10 min through the tail vein	Glutamine contribution to glutathione and pyrimidine nucleotide synthesis in chemotherapy-resistant AML tumors. *In vivo* and *in vitro* different usage of aspartate by AML cells	[17]
[^13^C]-lactate				
Infusion	Fasting of 16 h	[^13^C_2_]-lactate at 15 mg/kg/min by initial bolus followed by 0.2 mg/kg/min for 2 h through the tail vein under anesthesia	Contribution of circulating lactate to the TCA cycle and glutamine by detection of malate M+1 in subcutaneous mouse xenografts of NSCLC	[16]

NSCLC, Nonsmall cell lung carcinoma; PDX, patient-derived xenograft; PPP, pentose phosphate pathway; HBP, hexosamine biosynthetic pathway.

New insights into the metabolic reprograming of tumors have been acquired by continuously improving isotope tracing approaches. Broadly, typical experimental designs include the following: (i) food deprivation of animal models before tracer administration, (ii) tracer supplementation, (iii) collection of tissues and biofluids of interest (iv), metabolite extraction from samples, (v) analytical measurement of metabolites by GC/LC-MS, and (vi) chromatographic peak integration and data analysis (Figure 1, upper panel). Importantly, although ^13^C, ^15^N, and ^2^H stable isotopes occur naturally at a very low level, their natural abundance can impact the isotopic composition of metabolites and confound the labeling derived from the tracer. For instance, one of the most common isotopic tracing relies on ^13^C, which displays a natural abundance of 1.07%. Therefore, correcting the natural abundance of stable isotopes deserves attention when rigorously analyzing the fractional enrichment of downstream metabolites [15].

In this review, we will discuss how tracer supplementation impacts global physiology and how this might confound the interpretation of metabolic processes occurring in healthy and transformed tissues. Likewise, fasting before tracing supplementation can trigger an adaptive metabolism in mice; a topic that will be addressed to broaden our understanding of its potential impact on animal physiology and tumor metabolism. We will illustrate these two sections with metabolomic data from stable isotope tracing by discrete bolus administration in cancer mouse models. Complex scenarios that arise when metabolic reprograming is studied by isotope tracing in nutritional-restricted mice will be also covered.

## 2 Does *in vivo* tracer administration challenge physiological metabolism?

Studying *in vivo* cancer-specific metabolic pathways in animal models by stable isotope tracing is challenging at several steps of the procedure, including the very first step i.e., tracer delivery for optimal enrichment in tumor cells. To avoid substantial disruption of the physiological homeostasis upon tracer delivery, an isotopic enrichment equivalent to 10–30% of the total circulating pool of the tracer is recommended. This will enable downstream labeling patterns without excessive impact on the bloodstream concentration of the given metabolite [4]. To deliver a tracer, several administration methods have been successfully developed and optimized.

Performed on conscious mice, single or multiple discrete boluses by intraperitoneal (i.p) or intravenous (i.v) injections or gavage provoke intense and transient tracer boosts in the bloodstream that might complicate/hinder data analysis and interpretations [16,17]. The unnatural systemic metabolic effects caused by bolus include glucose spikes [16], likely causing increases in insulin secretion following each [U–^13^C_6_]-glucose injection. Repeated mice handling to deliver the tracer through discrete bolus also leads to acute stress responses which affect the whole-body metabolism [18]. Nevertheless, this tracer administration method is simple to perform and does not require the use of anesthesia, an advantage when considering the influence of anesthetics on cellular energy metabolism [19].

Continuous tracer infusion through the tail vein of sedated animals are advantageous because it allows a mild and continuous tracer delivery to achieve a stable concentration permitting robust evaluation of steady-state labeling of metabolic pathways in tissues of interest. Compared to this, tracer infusion through catheterization of the jugular vein on conscious immobile mice hold similar advantages to reach metabolic steady state, without the disadvantage related to the use of anesthetics. Importantly, infusion through a jugular vein catheter requires specialized surgical skills and expertise.

From a physiological point of view, tracer delivery by feeding animals with a solid or liquid diet containing the labeled nutrient is simple and advantageous – because the tracer is absorbed over time reaching physiological levels while not disturbing mouse feeding habits. Furthermore, it minimizes the metabolic response to stress induced by animal handling. Although this tracer delivery method is emerging as a promising strategy to perform *in vivo* stable isotope tracing, it is not often used because of limitations such as the strict control of the animal feeding behavior, long tracing periods, and a substantial economic outlay [20,21]. To date, the most commonly used procedures to deliver labeled nutrients are constant intravenous infusions and single or multiple discrete boluses (i.v, i. p, or gavage) (please refer to Table 1). As reviewed by Fernández-García et al. [7], the advantages and disadvantages of each method should be *a priori* understood to choose the best option according to the specific scientific question. Delivering an exogenous nutrient into an animal is not trivial, and depending on the administration method, it entails notable inherent technical limitations that may directly complicate the analysis of metabolomic data and interpretation of tumor metabolic phenotypes. Some of them have not been extensively covered in the literature, leaving a gap in our basic knowledge and much more space for improvement. Here, we focused on (i) the disruption of physiological homeostasis after bolus injections and (ii) the tissue-specific conversion of the tracer from one isotopologue to another and into its downstream metabolites.

### 2.1 Disruption of physiological homeostasis upon tracer administration

Although very few studies have reported results on this technical aspect, it is widely accepted that a discrete bolus of labeled nutrients causes intense and transient tracer peaks in the bloodstream as shown in mouse and human studies [16,17]. Such phenomenon has not been sufficiently described in the literature and deserves further attention because it can mislead the interpretation of tumor metabolic phenotypes. Therefore, we have explored the extent to which tracer delivery through multiple discrete boluses could stimulate physiological metabolism and cause global metabolic changes in plasma.

**Figure 2:**
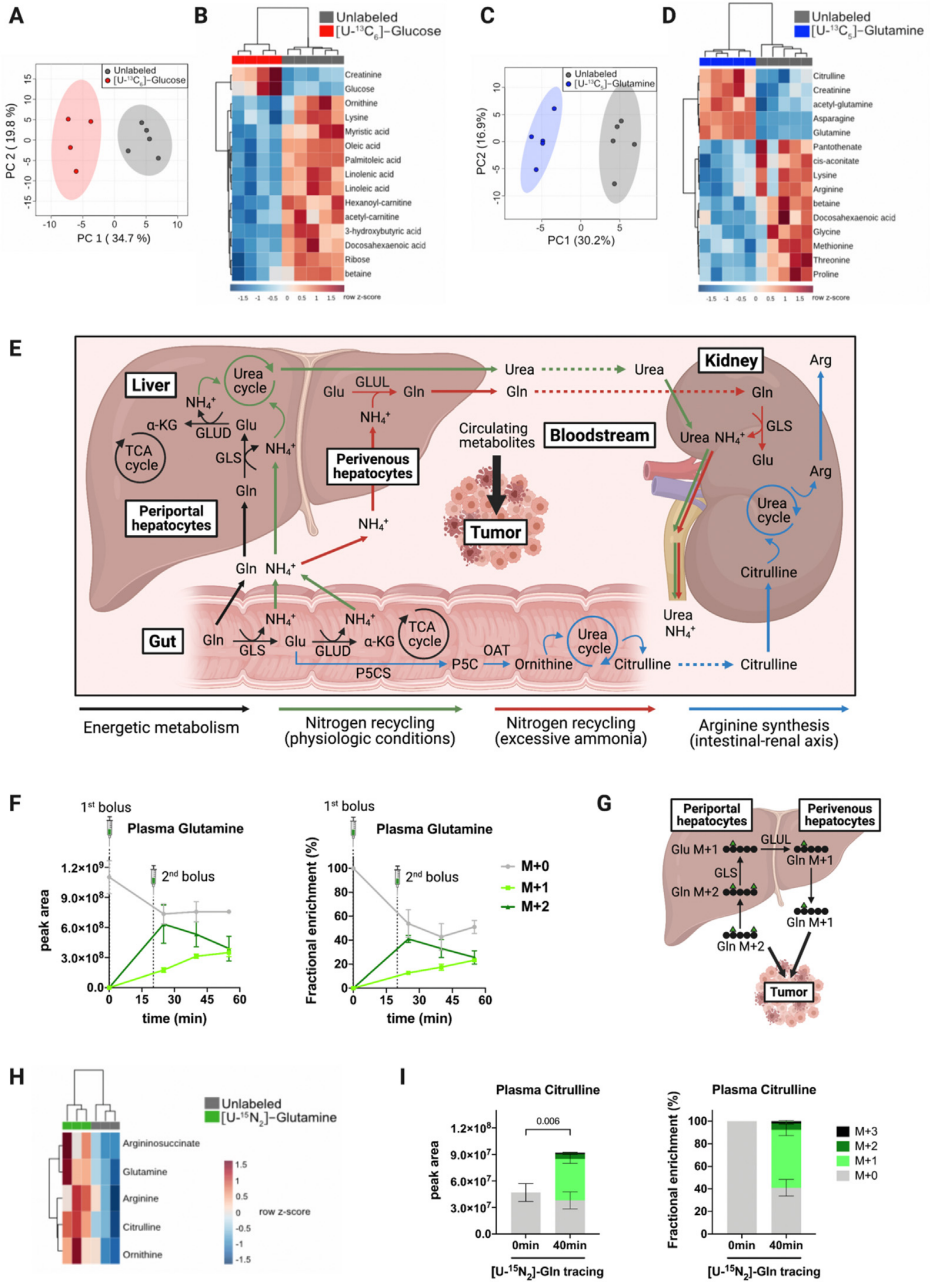
**Impact of tracer administration on physiological homeostasis and interorgan exchange fluxes. A,C.** Principal component analysis (PCA) plots of the circulating metabolome from tumor-bearing mice traced with [U–^13^C_6_]-glucose (n = 4) and [U–^13^C_5_]-glutamine (n = 5) *versus* unlabeled control mice (n = 5), respectively. The clustering has been performed based on 97 metabolites that were detectable and quantifiable in plasma with the LC-MS method used. **B,D.** Heatmap with the top 15 discriminant metabolites led to the clustering showed in panels A and C, respectively. Mice were intraperitoneally injected with two boluses separated by a 20-min interval of [U–^13^C_6_]-glucose (1 g/kg) and [U–^13^C_5_]-glutamine (0.3 g/kg). Blood was collected from the tail vein, 40 min after the first tracer injection. **E.** Schematic representation of the main glutamine interorgan exchange fluxes occurring in mammals and their impact on circulating metabolites that can be further taken up by tumors. **F.** Kinetics of M+0, M+1, and M+2 glutamine levels in plasma on [U–^15^N_2_]-glutamine tracing. Results are shown as peak areas (left panel) and fractional enrichment (right panel). Tumor-free mice were intraperitoneally injected with two boluses separated by a 20-min interval of [U–^15^N_2_]-glutamine (0.3 g/kg) and blood was collected from the tail vein at 0 min (n = 3), 25 min (n = 3), 40 min (n = 3), and 55 min (n = 3) after the first tracer injection. **G.** Schematic representation of the hypothetical conversion of circulating glutamine M+2 into glutamine M+1 on [U–^15^N_2_]-glutamine tracing. **H.** Heatmap representing changes in glutamine, argininosuccinate, arginine, citrulline, and ornithine levels in the plasma of tumor-free mice after 40 min of [U–^15^N_2_]-glutamine tracing (n = 3) compared to unlabeled mice (n = 3). **I.** Citrulline isotopologues in the plasma of tumor-free mice after 40 min of [U–^15^N_2_]-glutamine tracing (n = 3) *versus* unlabeled mice (n = 3). Results are shown as peak areas (left panel) and fractional enrichment (right panel). Results have been corrected for the presence of naturally occurring ^13^C stable isotopes using Metabolite AutoPlotter, a free online tool for metabolomics data processing [43]. Bars represent mean ± SD. Statistical differences were determined by two-tailed Student's t-test. α-KG, α-ketoglutarate; Arg, arginine; Gln, glutamine; Glu, glutamate; GLS, glutaminase; GLUD, glutamate dehydrogenase; GLUL, glutamate-ammonia ligase; NH_4_^+^, ammonium; OAT, ornithine aminotransferase; P5CS, pyrroline-5-carboxylate synthase; and TCA, tricarboxylic acid. Created with BioRender.com.

*In vivo* stable isotope tracing by two discrete bolus injections was performed on tumor-bearing mice. Glucose and glutamine are two of the most abundant metabolites in plasma and they play critical functions in the metabolism of tumors; therefore, we decided to perform *in vivo* tracing by two successive intraperitoneal injections at a 20 min interval with the following stable isotope tracers: [U–^13^C_6_]-glucose or [U–^13^C_5_]-glutamine. Carbon-labeled glucose delivery caused global changes in the circulating metabolome of tumor-bearing mice, as shown by principal component analysis (PCA) (Figure 2A). Interestingly, glucose and many free fatty acids appear among the top 15 significantly discriminant metabolites, which hint at variations in the systemic energetic metabolism upon discrete administration of exogenous glucose (Figure 2B). Similarly, global changes in the circulating metabolome of tumor-bearing mice were observed upon the delivery of carbon-labeled glutamine (Figure 2C). Glutamine, other amino acids, and urea cycle intermediates were among the top 15 significantly discriminant metabolites, suggesting alterations in the physiological metabolism of nitrogen upon discrete administration of exogenous glutamine (Figure 2D). These preliminary results indicate that discrete administration of exogenous nutritional sources can cause global changes in the circulating metabolome and possibly in the tumor metabolism, which could lead to misleading interpretations of tumor metabolic phenotypes.

Importantly, we cannot extrapolate to which extent plasma metabolic alterations induced by discrete bolus might impact the tumor metabolism. In cancer animal models, the nutrient composition of the plasma differs from that of the tumor interstitial fluid (TIF). Concentrations of a specific nutrient can either be increased, decreased, or unchanged in TIF compared to the plasma of a mouse with pancreatic cancer [22]. Depending on the nutrients modulated in plasma after tracer bolus, those alterations might not be mirrored in tumors. This will also be dictated by tumor-specific metabolic dependencies [10].

It could be argued that both the number of injections and the tracer doses we used are higher compared to other studies [23,24]. However, there are also examples in the literature where the total amount of [U–^13^C_6_]-glucose and [U–^13^C_5_]-glutamine injected is similar or even higher than what we have used [11,25,26]. Therefore, we encourage the scientific community studying cancer metabolism through *in vivo* stable isotope tracing to assess whether the delivery of a given tracer induces substantial changes not only in the tumor metabolome, but also in the circulating metabolome. Ideally, analysis of the tumor interstitial fluid metabolome would be even more informative. The experimental protocols thus could be optimized (e.g., lowering tracer concentration and/or doing single instead of multiple boluses) to minimize alterations in the physiological levels of the labeled source without compromising the isotopic enrichment required to track downstream metabolic reactions. As proposed by Yuan et al. [26], measuring glycemia throughout [U–^13^C_6_] glucose tracing experiments will be helpful to establish the optimal conditions. Similar real-time measurements of plasma glutamine concentration during tracer administration can also be considered using specific enzymatic analyzers.

### 2.2 Interorgan exchange fluxes of the tracer: a [U–^15^N]-glutamine case study

Owing to its role as an energetic substrate for mammalian tissues, glucose has been traditionally recognized as the main source of interorgan circulatory fluxes. However, recent elegant studies have shown that up to 37 metabolites are sufficiently concentrated in mouse plasma to substantially contribute to interorgan fluxes. Constant infusion is the best technical option to achieve the isotopic steady state, facilitating the interpretation of complex labeling patterns derived from the interorgan exchange of the tracer. Metabolic flux analysis (MFA), which aims to model complex metabolic networks only if steady-state isotopic labeling is achieved highlighted that circulating lactate has the highest circulatory flux and is a major carbon source for TCA cycle anaplerosis in most mammalian organs and certain tumor entities [27,28]. However, MFA is based on mathematical assumptions and simplifications of interconnected metabolic networks that sometimesan mislead interpretations [29].

It cannot be excluded that interorgan exchanges of labeled sources other than glucose-derived metabolites occur. When studying tumor metabolism, considering the interorgan exchange of the tracer is crucial – as the resulting tracer and its downstream metabolites may complicate plasma and tumor labeling patterns. This might be of high importance if occurring at the level of nutrients to which cancer cells are addicted, because it could bring confusing interpretations of tumor metabolic phenotypes. Here, we aimed to recapitulate the importance of metabolic compartmentalization and the main interorgan exchange fluxes occurring in mammals to sustain physiological homeostasis [27,[30], [31], [32]]. We will focus on the interorgan fluxes derived from glutamine metabolism (summarized in Figure 2E), the most abundant amino acid in plasma, and one of the most common substrates used to study tumor metabolism through *in vivo* stable isotope tracing (refer to Table 1).

Apart from its contribution to nucleotides and protein synthesis, glutamine is involved in many metabolic pathways to sustain the physiological functions of mammalian tissues [33]. Therefore, glutamine has a high interorgan exchange flux (Figure 2E). Dietary glutamine is absorbed by the gut, where it is subsequently deaminated into glutamate and α-ketoglutarate (α-KG) to fuel the TCA cycle and sustain the energetic demands of enterocytes and colonocytes [34]. Beyond the gut, glutaminolysis supports TCA cycle anaplerosis in almost every organ, with a particularly high contribution in the pancreas [27].

Glutamine carbons also fuel anabolic pathways [33] leading to production of glucose and glycolytic intermediates not only in gluconeogenic tissues (liver, kidney), but also in other organs, such as the pancreas [32]. In addition, the incorporation of glutamine to both glycolytic and TCA cycle intermediates provides a carbon skeleton for synthesis of several nonessential amino acids (NEAAs). Glutamine carbons also contribute to the synthesis of glutathione, proline, and arginine; the latest through a pathway that involves the intestinal–renal axis [[35], [36], [37]]. Briefly, glutamine-derived glutamate is converted into pyrroline-5-carboxylate (P5C) in the small intestine, which serves as a precursor for the synthesis of ornithine, an intermediate of the urea cycle. Therefore, glutamine fuels the urea cycle in the gut, which in turn releases citrulline into the portal vein that is further taken up by the kidney for *de novo* biosynthesis of arginine [38,39] (Figure 2E).

The amide and amine groups of glutamine substantially contribute to the physiological metabolism of nitrogen [33]. The amide group is incorporated into the synthesis of asparagine, nucleotides, hexosamines, and nicotinamide adenine dinucleotide (NAD). Similarly, the amine group serves for the synthesis of several NEAAs (e.g., aspartate, alanine, and serine) through glutamate transamination. The highly active nitrogen metabolism in mammalian tissues generates considerable amounts of ammonium, which is toxic. Therefore, mammalian organs (mainly the liver and the kidney) act in concert to recycle ammonium under diverse physiological conditions [39,40].

Liver zonation regulates ammonium recycling through compartmentalization of glutamine metabolism (Figure 2E). Under physiological conditions, periportal hepatocytes extract glutamine from the portal vein and subsequently catabolize it into glutamate and α-KG. Glutaminolysis in periportal hepatocytes generates a carbon skeleton to fuel the TCA cycle and produce two ammonium molecules that are recycled as urea by the urea cycle [39]. The urea produced in periportal hepatocytes is then released into the bloodstream and transported to the kidneys, where it is eliminated through the urine. The liver is the only mammalian organ with a full urea cycle, and therefore, it also recycles ammonium derived from the nitrogen metabolism of other tissues. In perivenous hepatocytes, glutamate generated in periportal hepatocytes is taken up and converted into glutamine through the recycling of ammonium. When ammonium is excessively concentrated in plasma, this glutamine–glutamate–glutamine cycle in the liver is enhanced to maximize its recycling [39,41,42].

Physiological glutamine interorgan exchanges can be illustrated with *in vivo* [U–^15^N_2_]-glutamine tracing, which we performed in tumor-free mice through two discrete bolus injections. Importantly, a transient equilibrium between labeled and unlabeled glutamine was reached at least 30 min after the second bolus of [U–^15^N_2_]-glutamine. Nevertheless, a switch from M+2 to M+1 glutamine (the latest being the main circulating isotopologue) occurred in a time-dependent manner (Figure 2F) [43]. Such an unexpected and quick phenomenon (25 min after the first bolus injection) suggests a tissue-specific conversion of [U–^15^N_2_]-glutamine through the glutamine–glutamate–glutamine cycle between periportal and perivenous hepatocytes (Figure 2E, G). As the excessive intake of dietary protein or amino acids has been shown to increase renal ammonium excretion [40], we hypothesized that [U–^15^N_2_]-glutamine administration might stimulate glutaminolysis, and concomitantly, ammonium production. Therefore, this would activate ammonium recycling through the glutamine–glutamate–glutamine cycle in the liver, leading to the mix of circulating glutamine isotopologues that we observed.

Glutamine is highly taken up and metabolized by certain tumor types such as glioma and liver tumors [44,45]. Although not formally proved, it is likely that the presence of substantial glutamine in the bloodstream following two discrete [U–^15^N_2_]-glutamine boluses can alter the labeling patterns of glutamine addicted tumors. However, the position of the labeled nitrogen in circulating glutamine M+1 remains unresolved: Do we have a mix or only a specific ^15^N_1_-glutamine isotopomer (^15^N_1_-amine- and/or ^15^N_1_-amide-glutamine)? Since liquid chromatography coupled to tandem mass spectrometry (LC-MS-MS) was not performed, we cannot illustrate the isotopomer distribution of the glutamine M+1 isotopologue. Nevertheless, we hypothesized that conversion of glutamine M+2 into M+1 might occur in the liver (Figure 2F) [43]; thus the labeled nitrogen of glutamine M+1 should correspond to its amine-group (Figure 2G). In this case, the relative abundance of the metabolites that incorporate the ^15^N-amide-group of glutamine would be under-estimated; possibly leading to false conclusions. Normalization according to the percentage of plasma glutamine M+2 enrichment would be a solution to correct the loss of labeled amide. However, this requires the achievement of a circulating isotopic steady state, which is not reached through tracer administration by bolus (es).

Physiological [U–^15^N_2_]-glutamine interorgan exchanges can also lead to the presence of other circulating labeled metabolites. For instance, glutaminolysis would generate an excess of labeled ammonium in the bloodstream. Although ammonium is physiologically recycled in the liver and excreted by the kidney through urine, this does not account for complete elimination [40]. Therefore, in the context of cancer, circulating labeled ammonium might be taken up and metabolically recycled by tumors through ammonium assimilating enzymes [46]. Reinforcing the hypothesis of a dynamic nitrogen metabolism upon [U–^15^N_2_]-glutamine administration, we observed increased levels of all urea cycle intermediates in the plasma of mice after 40 min of tracing (maximal glutamine abundance) (Figure 2H). Furthermore, consistent with the physiological synthesis of arginine through the intestinal–renal axis (Figure 2E), total levels of plasma citrulline were significantly higher and substantial levels of labeled citrulline were detected in circulation after 40 min of [U–^15^N_2_]-glutamine tracing (Figure 2I) [43]. Further *in vivo* isotope tracing experiments are required to refine the proposed hypothetical mechanism of interorgan exchange that might occur after discrete bolus administration of nitrogen-labeled glutamine.

It is also important to mention that if instead of tracing [U–^15^N_2_]-glutamine we had traced [U–^13^C_5_]-glutamine, we would have never observed the fast time-dependent conversion of one glutamine isotopologue to another in the bloodstream – because the carbon skeleton is maintained throughout this physiological glutamine–glutamate–glutamine cycle (Figure 2G). Therefore, the choice of the tracer is an important factor to consider according to the biological model used and the specific scientific question raised, when designing *in vivo* stable isotope tracing protocols.

Whether glutamine interorgan exchange might occur following continuous infusion of [U–^15^N_2_]-glutamine still needs to be addressed. Nevertheless, it seems plausible that this phenomenon is likely to be exacerbated by discrete administration methods, which do not allow for a constant supply of the tracer and require injections of the tracer at high concentrations and/or multiple boluses (refer to Table 1).

Although we cannot extend our results to other tracers, the interorgan exchange of circulating nutrients other than glutamine has been already reported in mammals [[30], [31], [32]]. Whether the resulting labeled metabolites in the bloodstream can alter tumor labeling patterns might depend on the avidity of cancer cells for this given nutrient. Recently, a glucose–alanine cycle between tumor and liver has been reported in a zebrafish melanoma model [47]. After considering the potential impact of delivering a tracer by discrete bolus, optimization of experimental settings would help to minimize the isotopic labeling of the tracer derived from an interorgan exchange, as it naturally happens. In our case, shortening the [U–^15^N_2_]-glutamine tracing period partially prevented the conversion of glutamine M+2 into glutamine M+1 without impacting tracer enrichment in the bloodstream (Figure 2F). Other parameters such as the route of administration might influence plasma and tumor labeling patterns. For instance, the route used to administer ^13^C-labeled fructose has been shown to impact the way it is metabolized [48]. Therefore, it would be of interest to investigate whether delivering a given tracer through different discrete administration methods (oral, i. p, i. v) influences the outcome of the experiment. Finally, another parameter to consider is the fasting period that is usually performed before constant tracer infusions (refer to Table 1). However, the impact of fasting when performing *in vivo* stable isotope tracing has not been fully addressed and it will be considered in the following section.

## 3 To be fasted or not to be fasted? The paradigm of fasting in isotope tracing

Fasting of animal models before tracer administration is commonly included as part of most *in vivo* isotope tracing protocols despite any consensus. Fasting is expected to maximize tissue uptake of the labeled metabolite and minimize fluctuations in the concentration of plasma and tissue metabolites caused by variable feeding behaviors among animals fed *ad libitum*. This common practice might be analogous to the routine overnight fasting required in the clinic before measuring serum biochemical variables in humans (glycemia, cholesterol, for instance). Interestingly, changing this clinical standard to a more practical nonfasting blood sampling when measuring the lipidic profile has been effective for predictions of cardiovascular disease risk in humans [54].

In the case of *in vivo* stable isotope tracing, fasting is not intended to change animal metabolism, but to provide a basal postabsorptive metabolic state as has been described in humans [55]. Indeed, intraoperative [^13^U–C_6_]-glucose infusions for metabolomic analysis of resected tumors and biopsies from adults and children with different types of cancer have been performed under fasting as required by a surgical intervention [16,56,57]. In the context of cancer research on animal models, rigorous studies to experimentally determine the optimal fasting duration to achieve a basal metabolic state in mice have not been undertaken yet. Interestingly, in stable isotope tracing studies, mice commonly undergo fasting periods that last longer than the clinical standard for human blood sampling. Whether mice deprived of food for 8h and longer are just fasted or rather starved is to be determined. Beyond the fasting duration, mice are also commonly fasted during their active night cycle, a practice that disrupts their natural feeding-fasting rhythm.

Few recent studies have investigated metabolite fluxes in fasted and fed states by independently infusing several labeled metabolites. However, analysis of total metabolite levels in plasma and tissue beyond labeling enrichment has not been reported [31,32]. Here, we discuss (i) the major systemic effects of fasting in mice and (ii) the impact of fasting on the levels of tumoral metabolites upon discrete bolus injections of fully carbon-labeled glucose. The latter topic will be illustrated with our metabolomics and tracing data.

### 3.1 Adaptive metabolism to food deprivation in mammals

**Figure 3:**
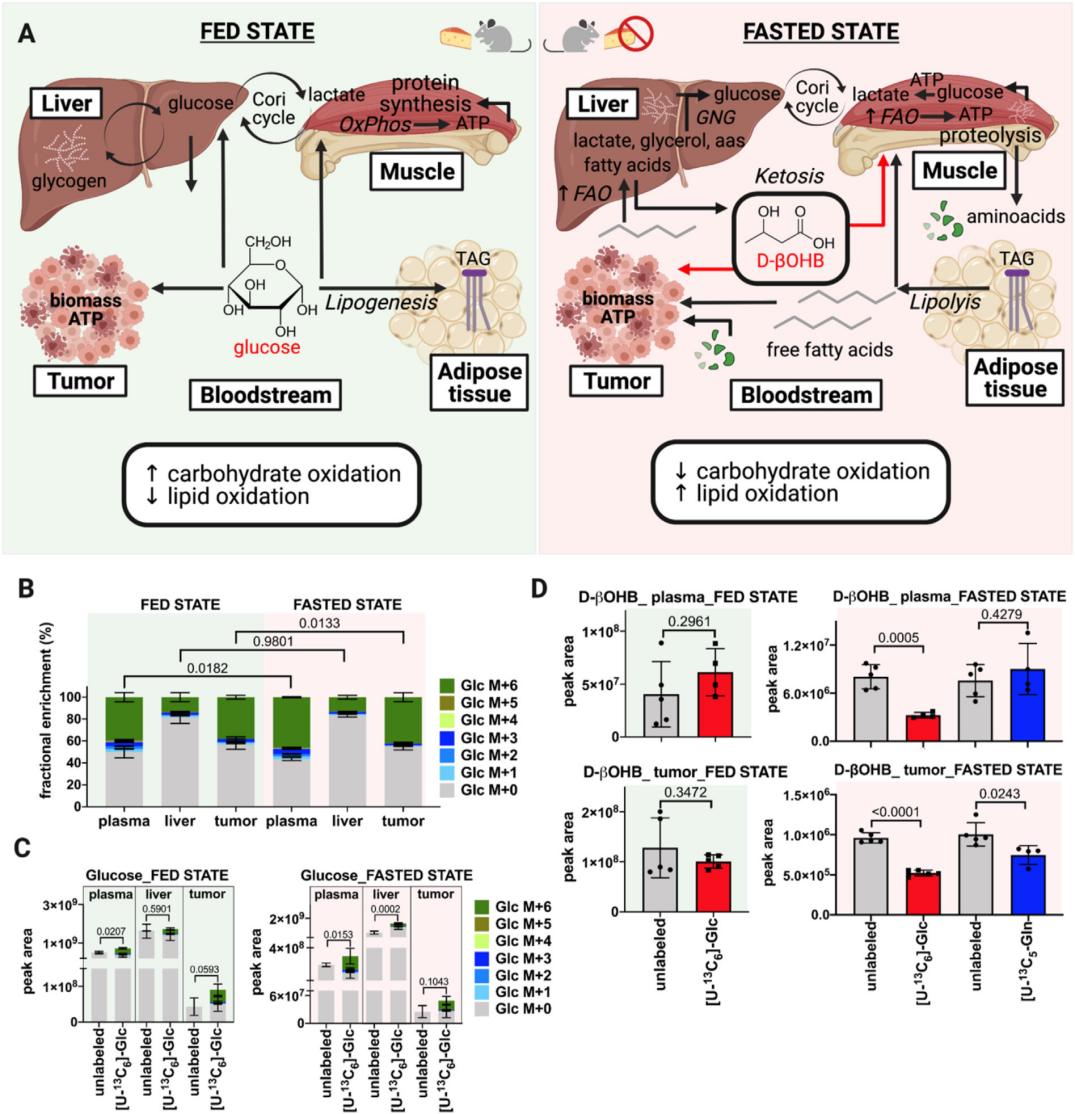
**Metabolic plasticity during feeding and fasting. A.** Schematic representation of the systemic metabolic phenomena occurring in fed and fasted states. Glycogen breakdown, gluconeogenesis, fatty acid release by lipolysis, and systemic switch from carbohydrate to lipid oxidation during the transit from fed to fasted states. Systemic changes impact tumoral metabolism depending on the availability of energetic substrates. **B.** Fractional enrichment of ^13^C-labeled glucose in plasma, liver, and tumor of fasted (n = 4–5) and fed mice (n = 4–5) on [U–^13^C_6_]-glucose administration. **C.** Peak area of glucose isotopologues in plasma, liver, and tumor of fasted (n = 4–5) and fed mice (n = 4–5) traced with [U–^13^C_6_]-glucose *versus* their respective unlabeled control mice (n = 4–5). [U–^13^C_6_]-glucose (1 g/kg) was administered by two intraperitoneal boluses separated by a 20-min interval in 3 h-fasted and fed tumor-bearing mice. Blood was collected from the tail vein 40 min after the first tracer injection. Results have been corrected for the presence of naturally occurring ^13^C stable isotopes using Metabolite AutoPlotter, a free online tool for metabolomics data processing [43]. **D.** Total levels of d-β-hydroxybutyrate in plasma and tumor of fed and fasted mice traced with [U–^13^C_6_]-glucose (1 g/kg) and [U–^13^C_5_]-glutamine (0.3 g/kg) *versus* their respective unlabeled control mice (n = 4–5). Bars represent mean ± SD. Statistical differences were determined by the two-tailed Student's t-test. OxPhos, oxidative phosphorylation; FAO, fatty acid oxidation; TAG, triglycerides; D-βOHB, d-β-hydroxybutyrate. Created with BioRender.com.

Metabolic plasticity in fed, postabsorptive, and fasted states allows organisms to adjust their metabolic demands to nutrient availability (Figure 3A). In humans, the smooth metabolic transition from fed to fasted state is regulated by insulin, glucagon, and other hormones. Major changes after two or three days of fasting are glucose and nonesterified fatty acid release into plasma because of hepatic glycogenolysis and lipolysis in adipose tissue [58]. When hepatic glycogen stores are depleted, gluconeogenesis remains as the main hepatic pathway producing circulating glucose [59]. In accordance with this adaptive metabolism in humans, mice undergoing diurnal fasting for 8 h use glycogenolysis and gluconeogenesis for synthesis of glycolytic intermediates in most organs [32]. Indeed, gluconeogenesis has been shown to contribute more than glycogenolysis to circulating glucose, whereas glycogen stands out as a major contributor of glycolytic intermediates in mice fasted for 8 h [32].

Mice fasted for 8 h display higher glycogen breakdown in most organs to fuel tissue energetic demands as compared to fed animals. Gluconeogenesis contribution to circulating glucose and TCA cycle intermediates also occur in the liver and extrahepatic tissues of mice fasted for 8 h. Among the gluconeogenic substrates, lactate and glycerol contribute the most to circulating glucose and the TCA cycle in tissues. Major differences between 8 h-fasted and refed mice come from increased glycerol usage for the production of circulating glucose in fasted animals. This correlates with a key metabolic feature of the fasted state, i.e., the catabolism of triglycerides producing free pools of glycerol for gluconeogenesis [32]. In mice fasted for 8.5 h, glycerol followed by alanine and fatty acids made a higher direct contribution to circulating glucose than in fed mice [31]. Glucose conversion to circulating lactate (the Cori cycle) for subsequent lactate oxidation by tissues has been described as the main flux dictating carbohydrate oxidation irrespective of 8 h-fasting and refeeding in mice [32]. Apart from the Cori cycle with a flux rate notably decreased in fasted mice, other major circulating metabolite fluxes are not significantly perturbated after food deprivation for 8 h [31]. Overall, when a steady-state labeling is reached after the infusion of tracers other than glucose and fructose, fasting does not significantly change animal tissue nutrients consumption [31]. Besides adipose tissue and muscle, minor changes in tissue nutrient usage occurred in fasted mice for 8 h. The analysis of the specific labeled nutrient contribution to tissue metabolites along with the determination of total metabolite levels in plasma and tissue will bring novel notions of systemic and organ-specific changes because due to fasting.

Carbohydrate oxidation from dietary glucose constitutes the main source of energy production upon feeding. Mice refed during the night after diurnal fasting have shown two-fold higher carbohydrate oxidation and glucose turnover from the circulation as compared to fasted animals [32]. In the fasted state, energetic demands are satisfied from different substrates as compared to the fed state. As the availability of dietary glucose drops over the fasting period, there is a gradual switch from carbohydrate to fatty acid oxidation. Fatty acid oxidation in the liver not only yields energy for hepatic demands, but also produces alternative fuels such as ketone species by ketogenesis [59]. Circulating ketone species are taken up by extra-hepatic tissues such as the brain, heart, and skeletal muscle for production of energy by terminal oxidation [60]. Indeed, the human circulating metabolome during starvation is characterized by high levels of ketone species, unsaturated long-chain fatty acids, and acylcarnitines along with ketogenic amino acids and their catabolites. Most of these metabolites reach significantly higher levels after two days of fasting in humans [58].

Mice fasted for 8.5 h during the day showed an augmented contribution of fatty acids to tissue TCA cycle intermediates as compared to fed animals. In addition, fatty acids were the major direct contributors to circulating D-β-hydroxybutyrate (D-βOHB), the most abundant ketone body. The direct contribution of this ketone to the tissue TCA cycle was higher in the fasted state. Thus, systemic carbohydrate and fat contribution to tissue TCA cycle stand out as the major metabolic phenomena differentiating fasted and fed states, respectively. Signs of this metabolic switch have been observed in mice fasted for 8.5 h [31]. Fasting, as a physiological challenge, elicits an evolutionarily conserved and adaptive metabolic mechanism characterized by a gradual switch in substrate usage to cope with energetic demands when dietary glucose becomes scarcer over time (Figure 3A).

### 3.2 Impact of fasting for *in vivo* stable isotope tracing on mouse physiology

Most of the stable isotope tracing studies in cancer mouse models in the previous years show variability in experimental setups with respect to fasting duration and timing (refer to Table 1). Several studies infusing glucose, glutamine, and lactate have reported fasting periods of 6 or 16 h [10,14,16,28,[50], [51], [52]]. For those studies where the fasting time was detailed, both diurnal and nocturnal fasting appeared as options. Despite the absence of consensus, overnight fasting for 16 h is the most common practice. Interestingly, one of the most recent tracing studies in healthy mice has reported that diurnal fasted animals for 8 h display a respiratory exchange ratio (RER) that decreased from 0.9 to 0.8 after 2 h of fasting. As a measure of carbon oxidation, RER was below 0.8 after 4 h of fasting onset, reflecting definitive fat burning [31]. Therefore, fasting for 16 h is expected to induce metabolic adaptations that rely on fatty acid oxidation for energy production.

Another critical aspect of fasting beyond its duration is associated with its daily timing. Since mice are nocturnal mammals consuming food mainly in their active night cycle, an imposed nocturnal fasting disrupts their natural feeding-fasting cycle. Under normal conditions, the systemic circadian clock couples brain and systemic signals with the organism behavior to anticipate and adapt nutrient availability to energetic demands along the day–night cycle. Food intake and digestion along with carbohydrate and fat oxidation, as well as daily oscillations of the local and global metabolism are transcriptionally and hormonally regulated by the circadian clock [61]. Environmental desynchronization of the feeding-fasting rhythm of mice with their natural wake–sleep cycle might lead to metabolic alterations [61,62], whereas reinforcing the natural circadian rhythm with night-restricted feeding and diurnal fasting provides better control of physiological metabolic parameters [62,63]. Mice undergoing diurnal or nocturnal fasting for only a day display perturbations in the circadian rhythmic expression of more than 80% of hepatic transcripts as compared to mice fed *ad libitum* [64]. Therefore, it is rational to argue that food deprivation for 16 h overnight before tracer administration entails not only a prolonged period of fasting, but also circadian metabolic perturbations. These potential physiological alterations should be experimentally determined and considered for *in vivo* stable isotope tracing experiments.

It is still unclear whether diurnal fasting impacts tracer enrichment in the bloodstream and global or tumor metabolism. Some suggestions can be made from our metabolomics studies. Subcutaneous tumor-bearing mice fasted in the morning for 3 h or fed *ad libitum* were supplemented with [U–^13^C_6_]-glucose by discrete boluses (Figure 3B) [43]. Tracer enrichment in the circulation was slightly and significantly lower in fed mice compared to fasted animals, although sufficiently high (40%) to detect labeling into downstream metabolites (Figure 3B) [43]. Whereas levels of [U-^13^C_6_]-glucose were not different in the liver, labeled glucose in tumors mimicked circulating levels of the tracer. Heterogeneity among samples did not stand out as a parameter that was dramatically changed between fasted and fed state. Thus, in terms of tracer enrichment, a diurnal fasting period of 3 h when performing glucose tracing by discrete boluses does not bring apparent benefits.

Administration of [U–^13^C_6_]-glucose increased total levels of glucose in plasma and tumor irrespective of fasted and fed state (Figure 3C) [43]. In the liver, total glucose levels upon tracer administration were increased in the fasted state, but not in fed mice despite similar enrichment of labeled glucose (Figure 3B) [43]. These higher levels of hepatic glucose correlate with higher levels of circulating glucose following its exogenous administration, and likely, with an augmented hepatic appetite for sugar upon its availability.

Total levels of circulating d-β-hydroxybutyrate (D-βOHB) were significantly decreased in fasted mice on supplementation with [U–^13^C_6_]-glucose, but not with [U–^13^C_5_]-glutamine (Figure 3D, upper panel). As glucose is the preferential energetic substrate at a systemic level, these results may indicate a ketogenic status that is not ameliorated by a less favorite source of energy, glutamine. Circulating levels of this ketone body did not change in fed mice irrespective of labeled glucose administration. In tumors, the abundance of D-βOHB was significantly decreased after labeled glucose supplementation and to a lesser extent upon labeled glutamine administration in fasted mice (Figure 3D, lower panel). No changes were observed in plasma and tumors of fed animals irrespective of labeled glucose administration. As a ketone body, D-βOHB could be synthesized at higher levels by its main producer, the liver, exported to the circulation and taken up by the tumor as an alternative energetic fuel in fasted mice. We cannot exclude that D-βOHB could also be produced at a higher extent by the tumor itself [60]. Irrespective of the source of this ketone body, reduced levels of intratumoral D-βOHB in fasted mice are likely associated with the exogenous administration of labeled glucose and glutamine. Therefore, fasting might impact tumoral metabolism by favoring the use of alternative energetic fuels such as ketones due to food deprivation. Even though tracer supplementation decreased intratumoral levels of D-βOHB to different degrees depending on the labeled nutrient exogenously administrated, terminal oxidation of D-βOHB through oxidative phosphorylation might still occur in fasted mice upon tracing as compared to fed animals. Tracing the fate of labeled carbons in fasted mice might partially reflect a gradual switch from ketone to tracer oxidation in tumors rather than a basal metabolic usage of the labeled nutrient by transformed tissues.

Metabolic alterations associated with fasting and subsequent tracer supplementation by discrete bolus cannot be ruled out. Beyond tracer supplementation-associated disturbances of the animal physiology, nonoptimized fasting periods may contribute to systemic and tissue-specific metabolic alterations. We encourage, as a rational alternative, (i) to perform *in vivo* stable isotope tracing experiments in fasted and fed mice to determine the optimal conditions in a cancer animal model of interest. *In vivo* tracing without prior fasting would not only be experimentally simpler, but also advantageous. If fasting is experimentally proven to be beneficial for *in vivo* tracing in cancer animal models, we suggest (ii) diurnal and short-term food restriction in mice.

Considering that fasting might impact animal physiology and stable isotope tracing, it is intriguing to consider whether tracing in mice depleted of certain nutrients will lead to confounding interpretations. Such a complex scenario will be illustrated in the next section, focusing on mice under amino acid-restricted diets or amino acid-depleting antimetabolic drugs.

## 4 Impact of amino acid restriction approaches on *in vivo* stable isotope tracing

Amino acids (AAs) not only serve as building blocks for protein synthesis, but also provide carbons and nitrogen atoms for anabolic reactions, energy production, regulation of the redox balance as well as epigenetic and post-transcriptional gene expression. Therefore, beyond their widely known glucose dependence, it is now well-established that cancer cells are also addicted to nonessential amino acids (NEAAs) to sustain tumor growth [65,66]. As a consequence, the classification of AAs as essential and nonessential does not properly reflect tumor dependencies and several NEAAs have been reclassified as conditionally essential in the context of cancer [67,68].

The AA requirements of cancer cells are dependent on several factors irrespective of their oncogenic mutations. However, both extracellular (i.e., in the TME) and intracellular AA availability dictate the dependency of tumors for certain AAs. In this sense, some tumors are dependent on *de novo* biosynthesis of the NEAAs that are present at very low concentrations in plasma, such as aspartate [69,70]. Similarly, some AAs (e.g., glutamine, and serine) are spatially depleted within the TME of certain cancer types [22], rendering tumors dependent on *de novo* biosynthesis [71] or extracellular protein scavenging [72,73]. In addition, conditionally essential AAs can also be *de novo* synthesized at insufficient concentrations to satisfy tumor needs, which partly explains the avidity of cancer cells for exogenous sources of such AAs. Moreover, some tumors are auxotrophic for certain NEAAs since epigenetic modifications suppress the expression of key metabolic enzymes involved in their *de novo* biosynthesis. For instance, the gene encoding for the asparagine synthetase (ASNS) enzyme is commonly silenced in acute lymphoblastic leukemia (ALL), rendering ALL cells auxotrophic for asparagine [74]. Similarly, several cancers are deficient for the urea cycle enzyme, argininosuccinate synthase (ASS1), which appears to be a metabolic advantage by diverting aspartate into *de novo* pyrimidine synthesis [75], but renders ASS1-deficient cancer cells auxotrophic for arginine [76].

Targeting the addiction of tumors for certain AAs appears to be a promising anticancer therapeutic strategy. In this context, AA dietary modifications are emerging as a potential approach to exploit the AA dependencies of tumors to enhance anticancer therapies [77,78]. For instance, tumor growth inhibition and sensitization to chemo- and radiotherapy have been observed in several mouse cancer mouse models fed a low-methionine diet [79]. Interestingly, dietary restriction of certain NEAAs has also shown antitumoral effects. For instance, serine/glycine-free diets have been efficient in reducing tumor growth in several xenografts and autochthonous mouse cancer models driven by different mutations [80,81]. More recently, limiting asparagine bioavailability through dietary restriction has been shown to reduce the metastatic potential of an orthotopic breast cancer mouse model [82].

**Figure 4:**
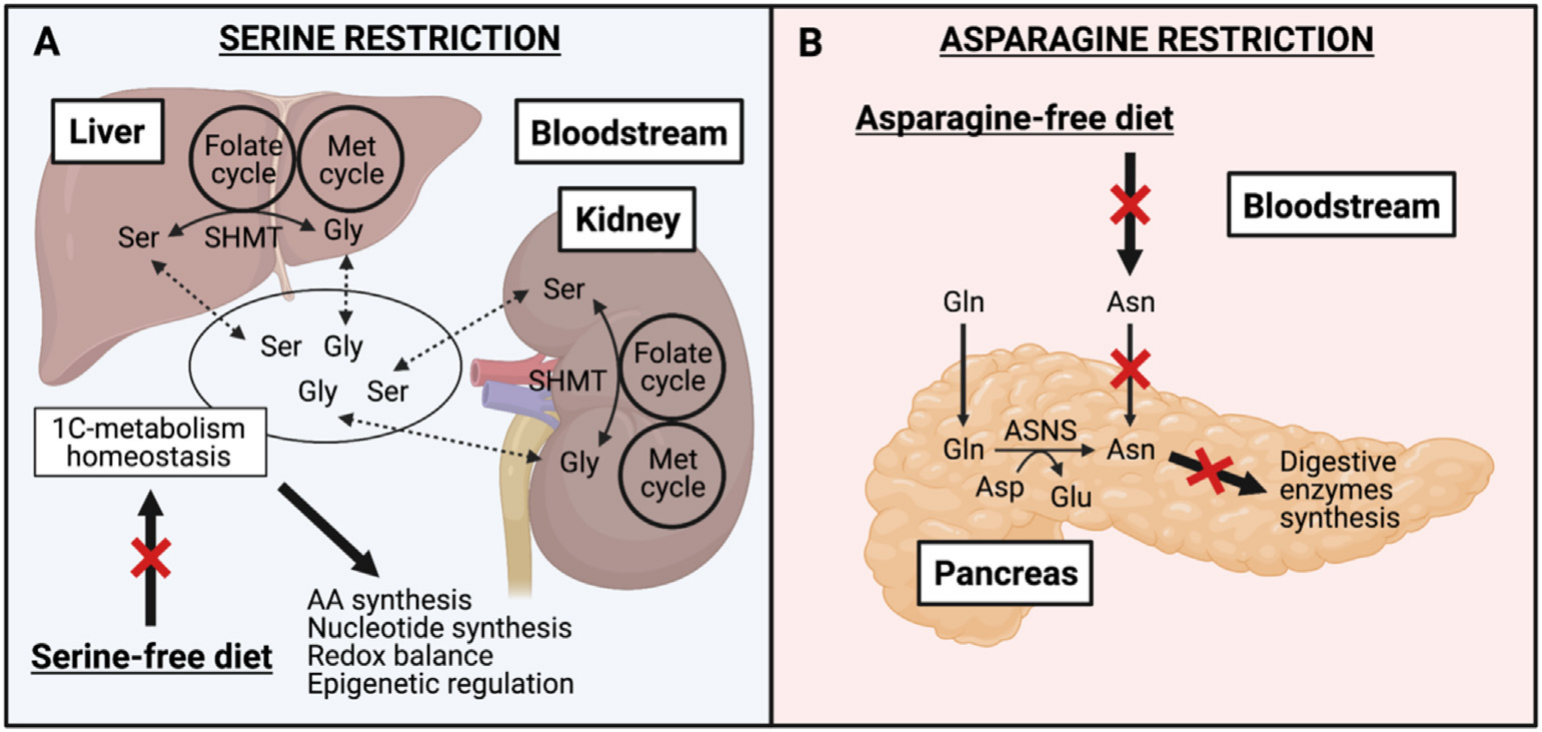
**Impact of systemic AA restriction in the physiological metabolism of healthy tissues**. **A.** Physiological functions of 1C-metabolism; the role of the liver and the kidney in the maintenance of its homeostasis and potential impact of serine-free diets. **B.** Pancreas dependence on asparagine uptake and *de novo* production for the synthesis of digestive enzymes and the potential impact of asparagine-free diets. 1C, one-carbon; AA, amino acid; Asn, asparagine; ASNS, asparagine synthetase; Asp, aspartate; Gln, glutamine; Glu, glutamate; Gly, glycine; Met, methionine; Ser, Serine; and SHMT, serine hydroxymethyltransferase. Created with BioRender.com.

Going a step further, it would be of great interest to study tumor metabolism in mouse cancer models under AA-restricted diets. Understanding how cancer cells adapt their metabolism to sustain tumor growth will help to identify and tackle the potential mechanisms of resistance occurring on AA restriction. In *in vitro* settings, stable isotope tracing appears to be a valuable tool to follow the metabolic adaptations of cancer cells deprived of specific AAs. [U–^13^C_6_]-glucose tracing *in vitro* has shown that *de novo* serine synthesis is induced by cancer cells under serine-deprived conditions. As a consequence, the combination of dietary serine restriction and pharmacological inhibition of phosphoglycerate dehydrogenase (PHGDH), a rate-limiting enzyme in the serine biosynthetic pathway, has shown antitumoral effects on mouse cancer models resistant to each treatment alone [81,83]. Indeed, *in vivo* [U–^13^C_6_]-glucose tracing has not been performed to directly prove increased *de novo* serine synthesis in tumors of mice fed with a serine-free diet. However, very few studies have applied *in vivo* stable isotope tracing to unravel tumor metabolism in animals upon AA restriction or any other antimetabolic treatment [17,84,85]. In addition, many of these studies have limited the use of *in vivo* stable isotope tracing to assess the efficacy of enzymatic inhibitors, without exploring potential tumor metabolic adaptations in response to a given antimetabolic treatment [[86], [87], [88]]. Based on the scarcity of these studies, we hypothesized that the physiological consequences of systemic AA deprivation might lead to a very complex isotopic scenario in the TME that challenges the interpretation of tumor labeling patterns. At a physiological level, it is not trivial to starve tumors of AAs without impacting the systemic metabolism. The depletion of circulating AAs disrupts the AA physiological homeostasis, which is dictated by the organ-specific metabolite turnover [30]. To illustrate this point, serine and glycine are highly interconverted both in the kidney and the liver, contributing to the systemic homeostasis of one-carbon metabolism, which supports important physiological processes (e.g., nucleotide and amino acid synthesis, redox balance, epigenetic regulation) [30,39,40,89,90] (Figure 4A). Despite being the organ expressing the highest levels of ASNS [[91], [92], [93]], the pancreas is addicted to exogenous sources of asparagine, a feature that is reflected by asparagine enrichment in digestive enzymes synthesized by pancreatic acinar cells as compared to the global proteome [94] (Figure 4B). Therefore, targeting tumor nutritional requirements using specific AA restriction strategies may also impact the metabolism of nontumoral tissues, especially those with specific AA metabolic functions, a phenomenon that might disturb the composition of the circulating metabolome. While plasma AA levels of tumor-bearing mice have been shown to remain highly constant even upon protein deprivation – partly owing to muscle atrophy to maintain a constant supply of EAAs in mammalian organs [78,95] – it is reasonable to argue that upon AA dietary restriction, the organism will undergo adaptative responses to sustain its physiologic metabolic functions. Based on the metabolic alterations observed on tracer administration (Figure 2), we hypothesized that performing *in vivo* stable isotope tracing on mouse cancer models fed with an AA-restricted diet might stimulate systemic adaptative responses leading to specific interorgan exchange and conversion of the labeled source. In this scenario, the interpretation of tumor labeling patterns would become particularly challenging, which might explain the lack of such studies in the literature.

## 5 Conclusions & perspectives

Despite the apparent difficulty of studying the metabolism of nutritional-restricted tumors by *in vivo* stable isotope tracing, we strongly believe that this is a necessary step to deeply understand the metabolic adaptations leading to treatment escape and tumor relapse. Therefore, we encourage the scientific community to address this hot topic. As previously described, careful selection of the tracer, optimization of its concentration, administration method, and duration of the tracing period will be key to designing protocols that can address specific scientific questions and obtain interpretable results. Thus, an exhaustive bibliographic study of the physiological adaptative responses occurring upon restriction of specific metabolites followed by an experimental optimization in the cancer animal model of interest will be required. Importantly, most tumor entities are composed of various cell types including cancer cells (evolving in hypoxic or oxygenated areas) and stromal cells (fibroblasts, immune, and endothelial cells). There is extensive evidence of cross-talk between cancer cells and stromal cells to sustain tumor metabolism [23,[96], [97], [98], [99], [100]]. Consequently, each cell-specific population contributes to the metabolic profile of the tumor *in vivo* [101,102]. When performing *in vivo* stable isotope tracing, these different populations might metabolize tracer, providing labeled metabolites independent of the circulation, another complex scenario that might resemble the interorgan exchange of labeled nutrients. Determining the metabolic features of cancer cells and different cell populations within the TME have been challenging till now; because the time needed to isolate the different cell populations is incompatible with the stability of the metabolites that have a rapid turnover [96]. Tracing labeled nutrients (for instance, [U–^13^C_6_]-glucose) into macromolecules with a lower turnover than metabolites has been proposed as an elegant solution to dissect cell-type specific metabolism in pancreatic adenocarcinomas [103].

## Funding sources

This project has received funding from the European Union’s Horizon 2020 research and innovation program under the Marie Skłodowska-Curie grant agreement No 766214 (META-CAN), by La Ligue Contre le Cancer, le Cancéropole PACA, l’Inserm and the Agence Nationale de la Recherche (LABEX SIGNALIFE ANR-11- LABX-0028-01). JC obtained a grant from la Fondation ARC.
